# Discourse type effects on EFL listening comprehension: cognitive load and metacognitive strategy shifts across scientific and literary texts

**DOI:** 10.1186/s40359-026-04684-2

**Published:** 2026-05-13

**Authors:** Mohamed Mekheimer

**Affiliations:** Faculty of Education, Beni-Suef University, Beni-Suef, Egypt

**Keywords:** EFL, L2 listening comprehension, Discourse type, Cognitive load, Metacognitive strategies, Listening anxiety, Individual differences

## Abstract

**Background:**

Discourse type can alter the demands placed on L2 listeners by changing coherence, rhetorical density, and the balance between meaning-driven and form-driven processing. Building on the Information-Rich Discourse Hypothesis, this study examines whether an information-rich scientific text and a rhetorically complex literary text produce different patterns of listening comprehension, perceived effort, and metacognitive strategy use among EFL learners.

**Methods:**

Sixty undergraduate EFL learners completed a within-subjects listening task involving two counterbalanced discourse conditions (scientific vs. literary). Outcomes included comprehension accuracy, processing speed, self-reported cognitive load, and self-reported use of top-down versus bottom-up metacognitive strategies. Analyses used paired comparisons and repeated-measures modeling that accounted for order and individual differences (listening anxiety and receptive vocabulary knowledge as a lexical-coverage proxy).

**Results:**

Listening to the information-rich scientific text resulted in significantly higher comprehension accuracy, faster processing, and lower cognitive load than listening to the rhetorically complex literary text. Strategy reports showed systematic adaptation across conditions, with greater reliance on top-down strategies in the scientific condition and greater reliance on bottom-up strategies in the literary condition. Individual differences were associated with variability in outcomes and strategy use; however, interpretations involving vocabulary are framed cautiously because the vocabulary measure reflects written receptive knowledge rather than phonological vocabulary access.

**Conclusion:**

Discourse type exerts robust effects on EFL listening performance, perceived effort, and strategic regulation. These findings support the Information-Rich Discourse Hypothesis and underscore that listening success depends on an interaction between discourse characteristics, learner profiles, and strategic processing.

**Supplementary Information:**

The online version contains supplementary material available at 10.1186/s40359-026-04684-2.

## Introduction

Listening comprehension in a second language (L2) is an effortful, time-sensitive process in which listeners must coordinate *bottom-up decoding* (segmenting the speech stream, recognizing words, parsing syntax) with *top-down inference* (using context, background knowledge, and prediction to build a coherent mental model) [1, 2]. Contemporary accounts converge on a central premise: comprehension success depends not only on listener proficiency but also on *how discourse properties shape processing demands* in real time [3]. When a listening text is coherent and well signposted, listeners can more readily anticipate upcoming content and integrate propositions efficiently, which supports meaning-focused processing and smoother comprehension [4–6]. In contrast, discourse with weaker signaling, heavier rhetorical density, or structural ambiguity can disrupt prediction and integration, forcing greater reliance on local decoding and increasing perceived effort [7, 8]. Thus, discourse type can alter both *what listeners understand* (accuracy) and *how efficiently they understand it* (processing speed), as well as the *mental effort* they experience (cognitive load) and the *strategies* they report using to regulate comprehension [2, 9].

This study is grounded in the Information-Rich Discourse Hypothesis, which proposes that listeners strategically reallocate processing resources depending on whether discourse prioritizes informational clarity/coherence or rhetorical form/interpretive openness [10]. At the same time, the present analysis does not treat this hypothesis as the only viable explanatory lens. Competing but partly compatible accounts - especially predictive-processing views, coherence-based discourse-processing models, and Cognitive Load Theory - also suggest that differences in signaling, predictability, and integration burden can shape listening outcomes.

On this broader reading, information-rich discourse (e.g., scientific exposition) should facilitate global meaning construction because its propositional structure is typically explicit and it supplies organizing cues (e.g., causal sequencing, definitions, exemplification, and summarizing conclusions). These properties strengthen predictability and discourse-level integration, allowing limited attentional resources to be directed toward higher-level semantic updating rather than prolonged form-level troubleshooting [2, 6, 10].

By contrast, rhetorically elaborate or structurally ambiguous discourse (e.g., literary/dramatic language characterized by figurativity, syntactic density, pragmatic indirection, or less linear topic development) is expected to increase interpretive uncertainty and raise local verification and repair demands, which can slow processing and elevate perceived effort [7, 10]. Accordingly, the present study interprets discourse effects primarily through the Information-Rich Discourse Hypothesis while also recognizing that the same pattern may be described in terms of predictive scaffolding, discourse coherence, and effortful resource allocation rather than a single exclusive mechanism.

Empirical work has increasingly shown that listening outcomes vary by *genre and discourse organization*, including differences in comprehension accuracy and the kinds of inferences listeners can sustain across texts [3, 6]. In the specific tradition motivating the present study [10], the authors provided foundational evidence that discourse type can yield distinct comprehension profiles and reported strategy patterns, broadly consistent with the *Information-Rich Discourse Hypothesis* [10].

However, two issues motivate a focused extension. First, the hypothesis has been tested most often with comparisons among discourse types that, despite rhetorical differences, remain goal-directed and communicatively engineered for comprehensibility (e.g., scientific explanation versus formal oratory). Second, fewer studies have examined whether the hypothesis holds when the “rhetorical” condition is not simply ornate but deliberately ambiguous and interpretively open, as in some literary or dramatic traditions. Such texts may violate conventional discourse expectations (e.g., cohesive topic progression, transparent pragmatic intent), creating a distinct challenge for L2 listeners that may alter not only performance but the strategic balance between prediction-driven comprehension and local decoding [7, 10].

Accordingly, the present study tests whether the discourse-type effects predicted by the Information-Rich Discourse Hypothesis generalize to a contrast between an *information-rich scientific* listening text and a *rhetorically complex literary/dramatic* listening text. The study focuses on three dependent-variable families explicitly: (a) *listening comprehension performance*, operationalized as accuracy and processing speed; (b) *self-reported cognitive load*, capturing perceived mental effort during listening; and (c) *metacognitive strategy use*, operationalized as the relative tendency to rely on top-down versus bottom-up regulation [9, 11, 12]. These outcomes are theoretically linked: if discourse properties change predictability and integration demands, then they should jointly affect performance, perceived effort, and strategy selection [2, 5, 9].

A second goal is to model learner-internal differences that are theoretically relevant to how discourse effects unfold. Listening anxiety is widely conceptualized as an affective constraint that can divert attentional resources and increase interference during online decoding and integration, particularly when input is less predictable and demands more monitoring and repair [13]. Vocabulary knowledge is also associated with listening comprehension because greater lexical coverage supports word recognition and proposition building; however, the interpretation of vocabulary effects depends on what the instrument actually measures. In the present study, the vocabulary test indexes written receptive vocabulary knowledge and is therefore best treated as a proxy for general lexical resources/coverage rather than as a direct measure of phonological lexical access or lexical automatization [14]. Accordingly, vocabulary is included as a covariate with a deliberately conservative interpretation, and any vocabulary-related inferences are framed as reflecting broader lexical resources rather than spoken-form processing efficiency.

The significance of this study is threefold. Theoretically, it provides a stringent test of the Information-Rich Discourse Hypothesis by extending it to a discourse contrast that is sharper in coherence and pragmatic transparency than prior comparisons [15]. Methodologically, it evaluates discourse effects using a multi-outcome approach (accuracy, speed, cognitive load, and strategy reports) rather than relying on comprehension scores alone, which clarifies whether discourse influences performance through perceived effort and strategic regulation [9, 12]. Pedagogically, it informs decisions about text selection, scaffolding, and strategy training for advanced EFL listening: if certain discourse types systematically elevate load and shift strategy demands, instructors can design supports that better match the processing profile elicited by the text [11, 16].

This study advances L2 listening research in three focused ways. Theoretically, it provides a stricter test of the Information-Rich Discourse Hypothesis by extending it from relatively goal-directed rhetorical contrasts to a sharper genre contrast (coherent scientific exposition vs. interpretively open literary/dramatic discourse), thereby clarifying how discourse affordances function as predictive scaffolding during online comprehension. Methodologically, it strengthens inference by using a within-subject design and covariate-adjusted sensitivity analyses via Linear Mixed Modeling (random intercepts; Satterthwaite degrees of freedom), which avoids unstable degrees of freedom and power loss associated with listwise deletion in over-parameterized general linear models. Pedagogically, it offers genre-sensitive guidance for scaffolding, material selection, and strategy training, especially for learners with higher anxiety or more limited lexical resources.

In short, the study clarifies when discourse structure *helps* listeners stabilize meaning efficiently and when rhetorical ambiguity *forces* costly repair—evidence that can guide both theory refinement and practical listening pedagogy.

### Research questions

1. How does discourse type (information-rich scientific vs. rhetorically complex literary/dramatic) affect EFL learners’ listening comprehension performance, measured by accuracy and processing speed?
2. What is the effect of discourse type on self-reported cognitive load during listening?
3. Does discourse type influence learners’ reported metacognitive listening strategies (top-down vs. bottom-up)?

### Hypotheses

1. Listening to the information-rich scientific discourse will yield higher comprehension accuracy and faster processing speed than listening to the rhetorically complex literary/dramatic discourse.
2. Listening to the information-rich discourse will yield lower self-reported cognitive load than listening to the rhetorical discourse.
3. Learners will report greater reliance on top-down strategies in the information-rich condition and greater reliance on bottom-up strategies in the rhetorical condition.

## Literature review

L2 listening comprehension emerges from an interaction between discourse properties, online processing constraints, and metacognitive regulation. Discourse type is therefore not a superficial label but a bundle of structural and pragmatic features—coherence, signaling, informational density, figurative load, and interpretive openness—that can shift predictability, integration demands, and repair requirements during continuous speech [2–6, 15, 17]. Building on this premise, the present review is organized around the study’s three outcome domains aligned to the research questions: (1) listening comprehension performance (accuracy and processing speed), (2) cognitive load/listening effort, and (3) metacognitive strategy use. A final subsection provides the rationale for modeling listening anxiety and vocabulary resources as covariates.

### Discourse type and L2 listening outcomes: accuracy and processing speed

A close anchor for the present study is a recent behavioral investigation that examined how *information density* and *rhetorical elaboration* shape EFL listening outcomes [10]. The core claim is that when discourse prioritizes content delivery through explicit organization and high coherence, listeners can allocate more resources to semantic integration and global meaning construction; when discourse foregrounds rhetorical form and stylistic elaboration, listeners face increased interpretive uncertainty and greater demands for local verification and repair [10]. Building on this logic, the present study extends prior work in three ways: (i) by operationalizing the genre contrast as information-rich scientific exposition versus rhetorically complex literary/dramatic discourse [10]; (ii) by modeling outcomes across comprehension success and efficiency (accuracy and processing time) as well as perceived effort (cognitive load) and metacognitive regulation (top-down vs. bottom-up emphasis) [2, 17–20]; and (iii) by estimating covariate-adjusted sensitivity models to evaluate whether discourse effects are robust to theoretically motivated learner differences in lexical resources and listening anxiety [13–15, 21–23].

A consistent finding across L2 listening research is that comprehension is sensitive to text type, discourse organization, and assessment design, rather than reflecting a single stable “listening ability” independent of input [3, 17, 24]. Genre and task format can shift observable performance by changing inferential demands and the extent to which listeners can exploit discourse organization [24]. Subsequent work indicates that expository and narrative texts often yield different comprehension profiles because they differ in propositional density, rhetorical moves, and predictability, affecting how readily listeners construct a coherent situation model and how efficiently they integrate propositions over time [3–6, 17]. These insights support the inclusion of processing speed alongside accuracy, because discourse differences may emerge not only in correctness but also in how quickly listeners can achieve stable interpretations under time pressure [17, 19].

A central mechanism linking discourse structure to performance is discourse signaling—lexical and structural cues that make macro-organization transparent (e.g., sequencing markers, contrast markers, summarizers). Signaling facilitates L2 listening by reducing the burden of organizing information and enabling more efficient global integration [6, 25]. Coherence-based and predictive-processing accounts converge on the idea that when discourse relations are explicit and topic development is stable, listeners can form stronger expectations and integrate meaning more smoothly, which benefits both accuracy and efficiency [2, 4–6]. Recent work on discourse comprehension likewise emphasizes that text/discourse features interact with individual and assessment factors, reinforcing the view that performance differences across discourse types reflect changes in processing opportunities and constraints rather than merely differences in “difficulty” [7, 17].

Measurement advances also support examining discourse effects through ecologically grounded performance indices. Dynamic approaches to speech comprehension assessment highlight that real-world listening ability involves continuous adaptation to changing demands, suggesting that efficiency-sensitive outcomes can capture meaningful discourse-driven variation beyond end-point accuracy alone [19]. In combination, this literature motivates the present contrast: an information-rich scientific discourse with high coherence and explicit structure should support more accurate and faster comprehension than a rhetorically complex literary/dramatic discourse that is less predictable and more interpretively open [7, 8, 10, 15, 17].

When discourse provides stronger coherence and clearer signaling, listeners are more likely to stabilize meaning earlier and integrate propositions efficiently; when discourse is rhetorically dense and less predictable, listeners face higher local decoding and repair demands that slow integration. Accordingly, the present study predicts higher comprehension accuracy and faster processing speed for the information-rich scientific discourse than for the rhetorically complex literary/dramatic discourse (H1).

### Discourse complexity and cognitive load: listening effort under time pressure

Listening imposes heavy demands on cognition because auditory input is transient and must be processed online: listeners must decode, store, and integrate information under time pressure [2, 18, 20]. Cognitive Load Theory conceptualizes perceived mental effort as arising from the interaction between task complexity and limited working-memory capacity, making it a useful framework for understanding why some listening conditions feel more effortful than others [18]. Contemporary accounts extend this perspective by framing listening effort as dynamic resource allocation across decoding, prediction, integration, and repair processes during continuous speech [20]. From this view, discourse properties can shape effort by altering predictability and the need for repair: discourse that supports stable expectations and transparent integration should reduce effort, whereas discourse that increases uncertainty and interpretive maintenance should elevate perceived load [7, 10, 20].

A growing body of experimental evidence demonstrates that listening effort is sensitive to linguistic and contextual challenges and can be indexed through convergent methods. Pupillometry studies, for instance, show that effort increases when listeners process challenging accent conditions, reflecting measurable changes in resource allocation [26]. Systematic review and meta-analytic evidence similarly indicates that adverse conditions such as noise reliably increase listening effort across measurement approaches, supporting effort as a robust construct rather than a purely subjective impression [27]. Although some of this work originates in speech perception and hearing science, its implication generalizes to L2 listening: increased uncertainty at the perceptual–linguistic interface tends to draw more resources and can constrain higher-level integration, especially under time pressure [2, 20, 27–29].

Discourse-level complexity should impose additional effort demands when texts contain syntactic density, rhetorical indirection, pragmatic opacity, or semantic ambiguity that requires maintaining competing interpretations and executing repairs [7, 10]. Evidence from bilingual language processing further suggests that increased cognitive load can impair working-memory updating and reduce prose recall quality, supporting the view that load can alter comprehension processes rather than merely accompany them [28]. Related neurocognitive evidence indicates that allocating processing resources during natural continuous speech is reflected in neural tracking patterns, linking effortful processing to measurable attention-related dynamics [20]. Collectively, these lines support the expectation that rhetorically complex literary/dramatic discourse will elicit higher perceived cognitive load than an information-rich scientific discourse, because the former is more likely to increase uncertainty, repair demands, and interpretive maintenance [7, 10, 20].

Effort rises when discourse increases uncertainty, repair, and interpretive maintenance under time pressure and falls when discourse provides an explicit organizational roadmap that reduces processing cost. Therefore, rhetorically complex literary/dramatic discourse is expected to elicit higher self-reported cognitive load than information-rich scientific discourse (H2).

### Discourse type and metacognitive regulation: shifts between top-down and bottom-up emphasis

Metacognition in L2 listening refers to learners’ capacity to plan, monitor, and evaluate comprehension, including decisions about when to prioritize global meaning construction (top-down) versus local decoding and form-based checks (bottom-up) [9, 12]. Research consistently links stronger metacognitive regulation to better listening outcomes and shows that strategy-focused instruction can improve comprehension—often with larger benefits among higher-proficiency learners—by strengthening planning, monitoring, and evaluation routines [11, 12, 30]. In particular, evidence from a quasi-experimental metacognitive intervention indicates that instruction can enhance listening comprehension and listening self-efficacy, with outcomes that may be more pronounced for higher-proficiency learners than for lower-proficiency learners [11]. Taken together, this supports treating metacognitive regulation as both outcome-relevant and task-responsive, while also motivating attention to proficiency-linked individual differences when interpreting discourse-contingent strategy shifts and any associated moderation patterns [11, 20].

At the same time, metacognitive regulation is not a fixed trait; it is responsive to task conditions. When discourse is coherent and information-rich, listeners can rely more on prediction, inferencing, and macro-level integration because stable cues support global meaning construction [2, 4–6]. When discourse is rhetorically complex and less predictable, listeners may shift toward strategies that emphasize local analysis, repeated checking, and repair to manage ambiguity and weaker signaling [7, 10]. This adaptive strategic shift is theoretically consistent with models in which listeners reallocate attention to maintain comprehension under changing uncertainty and processing demands [20].

Recent work strengthens the empirical basis for monitoring and repair as central components of listening strategy use. Real-time monitoring research indicates that listeners detect comprehension and non-comprehension episodes as listening unfolds, and that such monitoring can be documented systematically rather than inferred only from post-task self-report [31]. Complementary methodological work provides coding schemes for characterizing non-comprehension issues during listening, offering tools to describe how breakdowns arise and how listeners attempt to resolve them [32]. Intervention studies further show that metacognitive training can affect both listening comprehension and cognitive load, suggesting that strategic regulation may operate partly through more efficient allocation of processing resources rather than simply increasing the number of strategies reported [30]. Together, these findings support the expectation that discourse type will shape the relative emphasis of top-down and bottom-up strategy use by altering predictability, coherence, and the frequency of comprehension breakdowns [4–7, 10, 20, 31, 32].

If discourse affordances alter predictability and the need for repair, metacognitive regulation should adapt accordingly. Discourse that is coherent and strongly signaled is expected to support proactive, top-down meaning construction (prediction, inferencing, and global integration). By contrast, rhetorically complex and less predictable discourse should increase uncertainty and breakdown management, prompting greater reliance on reactive, bottom-up regulation (local verification, form-focused checking, and repair). On this basis, we predict stronger top-down strategy endorsement in the information-rich condition and stronger bottom-up endorsement in the rhetorical condition (H3).

### Individual differences as covariates: why vocabulary resources and listening anxiety matter

Discourse effects are unlikely to be uniform because listeners vary in cognitive and affective resources that shape online processing [2, 13, 17]. Listening anxiety has been conceptualized as a factor that can undermine comprehension by increasing worry and self-monitoring, consuming attentional resources, and interfering with efficient decoding and integration—effects that may be amplified under discourse conditions that heighten uncertainty and perceived loss of control [13]. Recent dynamic work further supports conceptualizing anxiety as a time-varying, person-specific factor in language learning, underscoring its plausibility as a meaningful source of variance in comprehension-related outcomes [33]. In discourse contrasts where one condition is structurally less predictable, anxiety-related interference may be especially relevant because uncertainty can increase monitoring costs and perceived effort [13, 20, 33].

Vocabulary resources are also associated with listening comprehension because lexical coverage supports proposition building and reduces reliance on compensatory inference when unknown items accumulate [14]. However, the construct assessed depends on the measurement instrument. In this study, vocabulary is indexed using the updated Vocabulary Levels Test (uVLT) [23], which measures written receptive vocabulary knowledge. It therefore functions most defensibly as a proxy for general lexical resources and coverage rather than as a direct assessment of phonological vocabulary access or lexical automatization in listening [14]. Convergent evidence from psycholinguistics indicates that stable individual differences in real-time word recognition under challenging listening conditions are linked to downstream outcomes, reinforcing the broader premise that variability in online lexical processing can shape comprehension under difficulty [21]. Additional work suggests that domain-general auditory processing precision can contribute to variability in L2 speech learning and processing, providing a principled basis for expecting processing-related individual differences to interact with input demands, even when not all components are directly measured in a given design [22, 35]. Related evidence also indicates that language-analytic ability, print exposure, memory, and complex-syntax comprehension contribute to individual variation in comprehension performance, further supporting the inclusion of learner-level covariates in the present analysis [36].

#### Rationale for covariate adjustment

Vocabulary resources and listening anxiety were modeled as covariates because both are theoretically relevant sources of variance in listening success and listening effort. Vocabulary resources (as indexed here) can reduce lexical gaps that would otherwise cascade into integration failure and heightened repair, whereas listening anxiety can consume attentional resources through worry and self-monitoring, increasing perceived effort and disrupting efficient integration under uncertainty [13, 14, 20]. Including these covariates in sensitivity analyses therefore tests whether discourse-type effects remain robust when accounting for learner resources and affective interference [2, 13, 17].

#### Construct-validity

Because the uVLT is a written receptive measure, any vocabulary-related effects are interpreted as reflecting general lexical resources and coverage rather than phonological lexical access or lexical automatization during speech processing [23].

### Research gap and present study

Existing research supports three converging claims: (a) discourse type and signaling influence listening performance and efficiency [3, 6, 17, 19, 22–25, 35]; (b) discourse properties shape effort allocation and perceived cognitive load during continuous speech processing [2, 18, 20, 26–29, 34]; and (c) metacognitive monitoring and strategy regulation are outcome-relevant and responsive to task conditions, including the management of comprehension breakdowns [9, 11, 12, 30–32]. Nevertheless, two gaps remain. First, evidence for the Information-Rich Discourse Hypothesis has been built largely on discourse contrasts where both conditions remain goal-directed and communicatively engineered for comprehensibility; its generalizability to a rhetorical condition characterized by deliberate ambiguity and disrupted coherence remains underexplored [10, 15]. Second, comparatively few studies examine discourse-type effects concurrently across accuracy, processing speed, cognitive load, and strategic adaptation within the same design while situating these outcomes within contemporary models of dynamic resource allocation and monitoring during continuous speech [20, 31, 32]. The present study addresses these gaps by contrasting information-rich scientific discourse with rhetorically complex literary/dramatic discourse and by testing aligned predictions across performance, effort, and strategy domains while situating individual differences as theoretically motivated sources of variance [2, 13–15, 17, 20–23, 33].

Discourse type is theorized to influence L2 listening by changing how much the input supports prediction and global integration versus forcing local verification and repair. We therefore measured accuracy and processing time to capture comprehension success and efficiency under time pressure. Because discourse-driven difficulty should be expressed as increased mental effort when predictability is weaker, we also measured self-reported cognitive load immediately after each listening task as a proximal indicator of processing cost. Finally, the hypothesis predicts a shift in cognitive priorities; accordingly, we measured metacognitive strategy emphasis (top-down vs. bottom-up) to index adaptive regulation in response to discourse affordances. Together, these three outcome families provide a coherent test of an input → processing → regulation pathway rather than isolated “extra” variables.

## Theoretical framework: an input–processing–regulation model of L2 listening

**Fig. 1:**
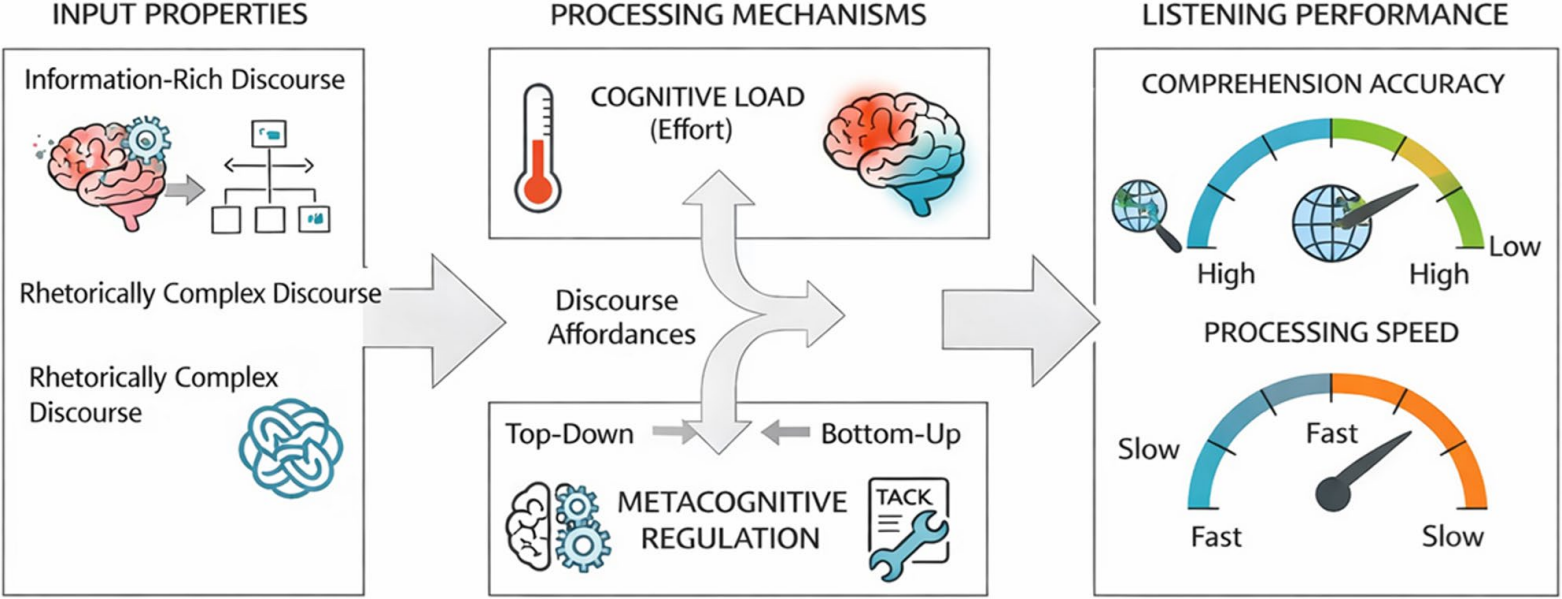
Theoretical framework of the input–processing–regulation model of L2 listening

This study adopts an Input-Processing-Regulation view of L2 listening in which discourse affordances (input properties) shape online resource allocation (processing effort) and trigger adaptive metacognitive regulation (strategic shifts). These mechanisms jointly determine observable listening performance - captured here as comprehension accuracy and processing speed. As shown in Fig. 1, discourse type is not treated as a genre label; rather, it is modeled as a continuum of predictive scaffolding that modulates uncertainty and repair demands during continuous speech comprehension [19, 26, 28].

At its core, the model assumes that comprehension depends on how quickly listeners can build and stabilize a coherent interpretation under time pressure. When discourse structure supports prediction and integration, meaning can stabilize earlier, freeing resources for higher-level integration. When discourse increases ambiguity and weakens structural cues, listeners must invest more resources in local decoding and repair, slowing stabilization and increasing effort. This account is intentionally compatible with alternative frameworks that emphasize predictive processing, discourse coherence, and cognitive-load management, so the model is presented as an integrative explanatory scaffold rather than a single closed theory.

### Discourse affordances as predictive scaffolding and their effects on performance (RQ1 / H1)

#### Key theoretical claim

Discourse type influences listening performance by altering the predictability of the input and the visibility of its macro-structure. Predictability is operationalized as the extent to which discourse provides a coherent organizational roadmap that supports anticipation, integration, and efficient updating during continuous speech [19, 26, 28].

#### Mechanism (input → performance)

Information-rich scientific discourse typically supplies stronger *predictive scaffolding* through explicit organizational cues—sequencing markers, causal connectors, definitional framing, and summarizers—which make discourse relations legible and reduce the organizational burden on the listener [19]. In contrast, rhetorically complex literary/dramatic discourse tends to increase interpretive openness (figurative meaning, pragmatic indirection, non-linear topic development, ambiguity), which weakens prediction, raises the probability of mis-commitment to an interpretation, and increases the need for repair and reinterpretation [28]. When interpretive openness is high, listeners may still reach correct answers, but doing so often requires more time because the system must repeatedly re-check lexical and structural hypotheses before committing to a stable meaning representation.

#### Prediction (H1)

Because information-rich discourse enables earlier *meaning stabilization*, it should produce higher comprehension accuracy and faster processing speed than rhetorically complex discourse, which increases uncertainty and repair demands and therefore slows stabilization [19, 26, 28]. Including processing speed as an outcome is consistent with approaches that treat real-world comprehension as an adaptive, time-sensitive capacity rather than a static accuracy-only endpoint [17].

### Cognitive load as the proximal processing mechanism (RQ2 / H2)

#### Key theoretical claim

Cognitive load (listening effort) is not merely a subjective impression; it reflects the *amount of processing effort* required to decode, predict, integrate, and repair meaning under time constraints. In this model, cognitive load functions as a *proximal driver* linking discourse affordances to performance outcomes [27–29, 34].

#### Mechanism (input → processing)

When discourse affordances align with listeners’ predictive capabilities—through stable coherence and clear structure—processing resources can be allocated efficiently: listeners spend less effort on reconstructing structure and more effort on integrating meaning [19, 28]. In contrast, rhetorically complex discourse increases the need for local decoding, hypothesis testing, and reinterpretation, which elevates processing demands and perceived effort. Evidence from listening-effort research shows that effort increases under challenging linguistic conditions and can be indexed through convergent methods such as pupillometry and multi-method syntheses, supporting the idea that increased uncertainty reliably draws additional resources [27, 29]. In bilingual processing contexts, higher cognitive load is also associated with weaker working-memory updating and reduced integration/recall quality, reinforcing the causal plausibility of the load pathway [34]. Neurocognitive evidence from natural speech comprehension further links comprehension quality to the allocation of processing resources during continuous speech, consistent with a resource-allocation interpretation of effort effects [31].

#### Prediction (H2)

Information-rich discourse should elicit *lower self-reported cognitive load* because its explicit structure functions as an organizational roadmap that offloads working memory demands and reduces the need for repair. Rhetorically complex discourse should elicit *higher cognitive load* because ambiguity and weaker predictability increase re-analysis and local decoding demands [27–29, 31, 34].

### Metacognitive regulation as adaptive strategy shifting (RQ3 / H3)

#### Key theoretical claim

Metacognition is modeled as a *dynamic regulatory system* that responds to the moment-by-moment state of comprehension. Rather than a static “strategy trait,” regulation is conceptualized as *adaptive control* over attention and processing emphasis—especially when listeners detect uncertainty or comprehension breakdown [21, 32, 33].

#### Mechanism (processing ↔ regulation)

The model distinguishes two broad regulatory emphases, not as mutually exclusive categories, but as *relative priorities* that shift with discourse affordances:

- Top-down regulation (proactive control): prediction, inferencing, macro-level integration, and using discourse structure to anticipate meaning. These processes are facilitated when discourse is coherent and signaled, because global structure can be exploited to guide interpretation efficiently [19, 28].
- Bottom-up regulation (reactive control): intensified attention to local decoding, word-level checking, syntactic parsing, and repair routines. These processes become more prominent when uncertainty rises—when listeners encounter ambiguity, unexpected transitions, or weak signaling and must verify or revise interpretations [21, 28, 33].

Recent research supports viewing monitoring and breakdown management as a central part of listening regulation. Real-time monitoring work shows that listeners track comprehension dynamically as listening unfolds, and that non-comprehension episodes can be observed systematically rather than treated as purely latent events [33]. Complementary methodological evidence offers structured ways to characterize non-comprehension issues, reinforcing the idea that breakdown and repair are patterned phenomena linked to input conditions [21]. Intervention research further indicates that metacognitive strengthening can improve comprehension and modulate cognitive load, which supports a model where regulation influences performance partly by shaping effort allocation and repair efficiency [32].

#### Prediction (H3)

A shift in discourse type should yield a corresponding shift in regulatory emphasis: information-rich discourse should promote *greater top-down regulation* (prediction/integration), whereas rhetorically complex discourse should force a shift toward *bottom-up checking and repair* to manage uncertainty and stabilize meaning [19, 21, 28, 32, 33].

Across the three hypotheses, the model predicts that *discourse affordances* alter the degree of predictive scaffolding available to the listener. Higher scaffolding (information-rich discourse) supports earlier meaning stabilization, lower effort, and greater top-down regulation, resulting in higher accuracy and faster processing speed. Lower scaffolding (rhetorically complex discourse) increases uncertainty and repair demands, raising cognitive load and shifting regulation toward bottom-up checking, which in turn slows and may reduce comprehension performance [17, 19, 21, 26–29, 31–34].

## Methods

### Research design

This study used a *within-subjects (repeated-measures) experimental design* to examine how discourse type shapes L2 listening outcomes. The within-participant independent variable was *Discourse Type* with two levels: *information-rich scientific/expository discourse (IR)* and *rhetorically complex literary/dramatic discourse (RH)*. Dependent variables were *listening comprehension accuracy* (0–12), *processing time* (seconds), *self-reported cognitive load*, and *metacognitive strategy emphasis* (top-down vs. bottom-up). To minimize order and fatigue effects, participants were randomly assigned to one of two *counterbalanced orders* (Order A: IR→RH; Order B: RH→IR).

### Participants

Participants were 60 undergraduate EFL majors at Beni-Suef University (45 female, 15 male; *M* age = 20.5, *SD* = 1.2). Based on departmental placement indicators, they were characterized as intermediate-to-advanced learners (CEFR B2–C1). Exclusion criteria included self-reported hearing impairment or diagnosed conditions affecting auditory processing. All participants provided informed consent and completed tasks individually in a controlled language-laboratory setting.

## Materials and instruments

### Listening stimuli and delivery

Two audio passages were developed to instantiate the discourse contrast while keeping length and rate comparable. The IR passage (“*The Unseen Guardian: Understanding Mangrove Ecosystems*”) was an expository text focusing on coastal engineering and carbon sequestration (581 words, 4:00, 145 words per minute). The RH passage (“*An Analysis of Hamlet’s Soliloquy*”) combined literary analysis with a dramatic segment from Shakespeare’s text (572 words, 4:00, 143 words per minute). Both recordings were produced by the same professional female narrator (standard British English accent) under studio conditions with consistent microphone and gain settings. Participants listened through high-fidelity headphones. No captions, transcripts, images, or other visual supports were provided.

### Listening comprehension assessment

Comprehension was assessed after each passage using a parallel 12-point test consisting of eight multiple-choice items (four literal, four inferential; 1 point each) and one global short-answer item (4 points). Multiple-choice items were used to standardize scoring and reduce construct-irrelevant variance from extended writing under time pressure. The short-answer prompt required participants to synthesize the central meaning in one sentence, targeting global integration rather than local recall.

Participants responded to the short-answer item in English (Arabic glosses of key technical terms were available to prevent vocabulary-related misunderstandings of the prompt). Short-answer responses were scored using an idea-unit rubric specifying required meaning components. Two independent raters scored the short-answer responses; interrater reliability was high (ICC = 0.92). Discrepancies were resolved through discussion to consensus.

### Processing time

Processing time was defined as the time (in seconds) required to complete the comprehension test after listening. Timing began automatically when the first comprehension item appeared and ended upon final submission. Timing was captured using the platform’s built-in timer/log (i.e., computer-recorded timestamps) under a standardized script.

### Self-reported cognitive load

Immediately after each comprehension test, perceived cognitive load was measured using a 3-item scale assessing mental effort, perceived difficulty, and frustration, rated on a 7-point scale (1 = very low/easy, 7 = very high/difficult). Internal consistency was high in the present sample (IR: α = 0.91; RH: α = 0.93). Items were presented in English with brief Arabic clarifications for technical wording where needed. Because the study was conducted in a regular university language-laboratory setting without access to eye-tracking, pupillometry, or other psychophysiological equipment, complementary objective effort indices were not collected; instead, the design prioritized immediate post-task self-report as a feasible and standardized indicator of perceived listening effort.

### Metacognitive strategy inventory (MSI)

Metacognitive strategy use was assessed with a 6-item text-specific inventory comprising two subscales: Top-down strategies (3 items; e.g., using background knowledge, focusing on main ideas) and Bottom-up strategies (3 items; e.g., focusing on individual words, analyzing sentence structure). Items were rated on a 5-point frequency scale (1 = never, 5 = very frequently). To avoid interrupting online processing during listening and to preserve comparability across the two passages, the MSI was administered after both listening tasks, with participants providing separate ratings for each passage (i.e., condition-specific retrospective reporting).

### Individual-difference covariates

Two covariates were collected to account for learner-level variance relevant to performance and effort outcomes:

1. Listening anxiety was measured using a 2-item scale assessing anxiety and confidence during demanding listening tasks (α = 0.89).
2. Vocabulary knowledge was assessed using the updated Vocabulary Levels Test covering the 2 K, 3 K, and 5 K frequency bands; the total percentage correct served as the vocabulary score. Because this instrument indexes *written receptive* vocabulary, it was treated as an indicator of general lexical resources/coverage rather than spoken-form access or automatization.

### Procedure

Data collection took place in a university language laboratory. Participants first completed the background profile, listening anxiety scale, and vocabulary test. They were then randomly assigned to one of the two counterbalanced orders. For each condition, participants (a) listened to the passage once through headphones, (b) completed the comprehension test immediately afterward (questions were presented after listening; no preview during listening), and (c) completed the cognitive load scale. After completing both listening conditions, participants completed the MSI twice—once for IR and once for RH—to yield condition-specific strategy ratings. All instructions and questionnaires were presented in English, with Arabic clarifications available for technical terms to ensure task comprehension without changing the response language.

### Data analysis

Primary discourse effects were examined using *paired-samples t-tests*, and associations among covariates and dependent variables were examined using *Pearson correlations*. To provide covariate-adjusted sensitivity checks and avoid power loss from listwise deletion, supplementary analyses used *linear mixed models (LMMs)* with a random intercept for participants and *Discourse Type* as a within-participant fixed effect. *Vocabulary* score and *listening anxiety* were entered as continuous covariates, and Discourse × Covariate interactions tested moderation. Degrees of freedom were estimated using the *Satterthwaite approximation*. In addition, Order (IR→RH vs. RH→IR) was inspected as an order-control factor (and as a Discourse × Order check) to confirm that counterbalancing did not account for the observed discourse effects. All analyses were conducted in SPSS (Version 28).

### Ethical considerations

Ethical approval was obtained from the Faculty of Education Institutional Review Board at Beni-Suef University (Approval Reference No. FoE-BSU-003-03-03-2025). Participation was voluntary, participants could withdraw at any time without penalty, and all data were anonymized and treated confidentially.

## Results

### Data analysis overview

All analyses were conducted in SPSS (Version 28). Because each participant completed both listening conditions (two observations per participant), the results are reported in two layers. First, paired-samples *t* tests quantify the within-participant discourse contrasts for comprehension outcomes and perceived effort (and the within-text strategy contrast). Second, covariate-adjusted sensitivity analyses use Linear Mixed Modeling (LMM) with a random intercept for participants, which retains the full sample and yields stable denominator degrees of freedom via the Satterthwaite approximation. Fixed effects included Discourse Type, Vocabulary Score, and Listening Anxiety; interaction terms (Discourse × Covariate) were included where the model was used to test moderation.

### Descriptive statistics

Table 1 presents descriptive statistics for the core dependent variables (accuracy, processing time, cognitive load) by discourse condition, alongside strategy indices and covariates.

**Table 1:** Descriptive statistics for main study variables (*N* = 60)

Measure	Information-rich discourse M (SD)	Rhetorical discourse M (SD)
Comprehension accuracy (0–12)	10.18 (1.53)	6.62 (2.29)
Processing time (seconds)	237.12 (32.93)	352.23 (40.47)
Cognitive load (1–7)	2.56 (0.74)	5.76 (0.57)
Top-down strategy use (1–5)	4.17 (0.47)	2.33 (0.49)
Bottom-up strategy use (1–5)	2.53 (0.55)	4.60 (0.32)

Strategy and covariate descriptives follow the study scoring rules (higher values indicate greater endorsement). Vocabulary Score: *M* = 77.27, *SD* = 10.29. Listening Anxiety: *M* = 2.63, *SD* = 0.67

### Associations among covariates and outcomes

To evaluate whether individual differences were meaningfully related to the dependent variables, Pearson correlations were inspected for Vocabulary Score and Listening Anxiety against performance, effort, and strategy indices. As shown in Table 2, Vocabulary Score correlated positively with comprehension accuracy and negatively with processing time and cognitive load, whereas Listening Anxiety correlated negatively with accuracy and positively with processing time and cognitive load. These association patterns indicate that both covariates relate systematically to the major outcome domains (performance, effort, and strategic regulation), supporting their inclusion as covariates in the sensitivity models.

**Table 2:** Correlations of covariates with primary outcomes and strategy indices (*N* = 60)

Variable	Accuracy (IR)	Accuracy (RH)	Time (IR)	Time (RH)	Load (IR)	Load (RH)	Top-down (IR)	Bottom-up (IR)	Top-down (RH)	Bottom-up (RH)
Vocabulary Score	0.621	0.979	−0.327	−0.571	−0.369	−0.471	0.643	−0.594	0.569	−0.607
Listening Anxiety	−0.951	−0.668	0.635	0.584	0.593	0.799	−0.683	0.610	−0.597	0.555

Values are Pearson’s *r*. IR = information-rich; RH = rhetorical. All shown coefficients are statistically significant at *p* < .001 in the correlation output

The strategy indices also showed extremely strong negative associations within each discourse condition (Top-down vs. Bottom-up), indicating that they behaved as near-reciprocal measures in this dataset (e.g., *r* = − .995 for the information-rich condition; *r* = − .991 for the rhetorical condition), which is consistent with the construction of the indices as complementary subscales and likely reflects shared measurement/scoring structure rather than two independent strategic capacities.

### RQ1: discourse type effects on listening performance (accuracy and speed)

Paired-samples *t* tests compared performance between the two discourse conditions. Accuracy was significantly higher for the information-rich discourse than for the rhetorical discourse, *t*(59) = 15.53, *p* < .001. Processing time was significantly shorter in the information-rich condition, *t*(59) = − 26.98, *p* < .001. The mean differences and confidence intervals are reported in Table 3, indicating a large and consistent performance advantage for the information-rich discourse across both correctness and efficiency.

**Table 3:** Paired-Samples t-Test Results for Discourse-Type Effects on Accuracy, Processing Time, and Cognitive Load

Comparison (A − B)	Mean difference	95% CI for mean difference	t	df	p
Accuracy: IR − RH	3.57	[3.11, 4.03]	15.53	59	< .001
Time: IR − RH	−115.12	[−123.66, −106.58]	−26.98	59	< .001
Cognitive load: IR − RH	−3.20	[−3.36, −3.03]	−39.65	59	< .001

*IR* Information-rich, *RH* Rhetorical. Negative mean differences for time and load indicate higher values (slower time or higher load) in the rhetorical condition

Paired-samples t tests compared performance between the two discourse conditions. Accuracy was significantly higher for the information-rich discourse than for the rhetorical discourse, t(59) = 15.53, *p* < .001. Processing time was significantly shorter in the information-rich condition, t(59) = -26.98, *p* < .001. The mean differences and confidence intervals are reported in Table 3, indicating a large and consistent performance advantage for the information-rich discourse across both correctness and efficiency. In practical terms, these were very large within-participant effects (Cohen’s dz = 2.00 for accuracy and 3.48 for processing time), suggesting that the discourse contrast was not only statistically reliable but also educationally meaningful within this task configuration.

Perceived cognitive load was significantly lower after listening to the information-rich discourse than after listening to the rhetorical discourse, *t*(59) = − 39.65, *p* < .001 (Table 3). The magnitude of the mean difference indicates that the rhetorical discourse imposed substantially greater subjective effort.

Perceived cognitive load was significantly lower after listening to the information-rich discourse than after listening to the rhetorical discourse, t(59) = -39.65, *p* < .001 (Table 3). The magnitude of the mean difference indicates that the rhetorical discourse imposed substantially greater subjective effort. The corresponding effect size was also very large (Cohen’s dz = 5.12), which underscores the practical relevance of the discourse-related effort gap rather than reducing the result to statistical significance alone.

### RQ3: Discourse type effects on metacognitive strategy emphasis

The strategy pattern showed a clear crossover consistent with discourse-driven adaptation: Top-down strategy endorsement was higher in the information-rich condition, whereas Bottom-up strategy endorsement was higher in the rhetorical condition (Table 1). Within each discourse condition, the two strategy indices also differed sharply (information-rich: Top-down > Bottom-up; rhetorical: Bottom-up > Top-down), consistent with the near-reciprocal relationship observed in the correlation matrix. In combination, these correlations support the inclusion of Vocabulary Score and Listening Anxiety as covariates across performance, effort, and strategy indices.

The strategy pattern showed a clear crossover consistent with discourse-driven adaptation: Top-down strategy endorsement was higher in the information-rich condition, whereas Bottom-up strategy endorsement was higher in the rhetorical condition (Table 1). Within each discourse condition, the two strategy indices also differed sharply (information-rich: Top-down > Bottom-up; rhetorical: Bottom-up > Top-down), consistent with the near-reciprocal relationship observed in the correlation matrix. Although these strategy contrasts are descriptive rather than inference-tested with a separate paired-effect estimate in this section, the size of the mean separation across conditions indicates a practically substantial re-weighting of strategic emphasis rather than a trivial shift in self-report tendencies.

To evaluate whether the discourse effects remained when accounting for learner differences, LMM sensitivity models were estimated with a random intercept for participants. Discourse Type was entered as a fixed within-participant factor; Vocabulary Score and Listening Anxiety were entered as continuous covariates, with Discourse × Covariate interactions included to test moderation. Across outcomes, the denominator *df* remained stable (approximately 57–59), indicating that the covariate-adjusted inferences were based on the full sample rather than a reduced subset.

For comprehension accuracy (Table 4), the main effect of Discourse Type remained significant, and both Vocabulary Score and Listening Anxiety explained substantial additional variance. Neither Discourse × Vocabulary nor Discourse × Anxiety was significant for accuracy, indicating that the discourse advantage was broadly consistent across levels of vocabulary and anxiety in this model.

**Table 4:** Linear mixed model fixed effects for comprehension accuracy (*N* = 60)

Source	Numerator df	Denominator df	F	*p*
Intercept	1	57.12	22.41	< 0.001
Discourse Type	1	58.05	42.89	< 0.001
Vocabulary Score	1	57.12	115.34	< 0.001
Listening Anxiety	1	57.12	89.12	< 0.001
Discourse × Vocabulary	1	58.05	2.45	0.123
Discourse × Anxiety	1	58.05	1.12	0.295

For cognitive load (Table 5), the main effect of Discourse Type remained significant when covariates were included. Vocabulary Score and Listening Anxiety were also significant predictors of perceived effort. The Discourse × Anxiety interaction reached significance, indicating that the discourse-related change in reported effort differed by anxiety level.

**Table 5:** Linear mixed model fixed effects for cognitive load (*N* = 60)

Source	Numerator df	Denominator df	F	*p*
Intercept	1	57.24	45.39	< 0.001
Discourse Type	1	58.11	19.29	< 0.001
Vocabulary Score	1	57.24	11.87	0.001
Listening Anxiety	1	57.24	102.91	< 0.001
Discourse × Vocabulary	1	58.11	0.84	0.362
Discourse × Anxiety	1	58.11	4.88	0.031

For Top-down strategy use (Table 6), Discourse Type was a strong predictor, with higher top-down endorsement in the information-rich condition. Both Vocabulary Score and Listening Anxiety were significant covariates, and both Discourse × Vocabulary and Discourse × Anxiety interactions were significant, indicating moderation of the discourse-driven strategy shift.

**Table 6:** Linear mixed model fixed effects for top-down strategy use (*N* = 60)

Source	Numerator df	Denominator df	F	*p*
Intercept	1	57.08	24.25	< 0.001
Discourse Type	1	58.02	265.26	< 0.001
Vocabulary Score	1	57.08	63.15	< 0.001
Listening Anxiety	1	57.08	70.26	< 0.001
Discourse × Vocabulary	1	58.02	113.01	< 0.001
Discourse × Anxiety	1	58.02	98.68	< 0.001

The Bottom-up sensitivity model (Table 7) showed a complementary pattern: Discourse Type strongly predicted bottom-up endorsement, with higher bottom-up processing reported in the rhetorical condition. Both Discourse × Vocabulary and Discourse × Anxiety interactions were significant, indicating that the magnitude of bottom-up shifting in response to rhetorical discourse varied systematically with learner differences.

**Table 7:** Linear mixed model fixed effects for bottom-up strategy use (*N* = 60)

Source	Numerator df	Denominator df	F	*p*
Intercept	1	57.31	28.95	< 0.001
Discourse Type	1	58.00	248.86	< 0.001
Vocabulary Score	1	57.31	83.13	< 0.001
Listening Anxiety	1	57.31	77.77	< 0.001
Discourse × Vocabulary	1	58.00	111.17	< 0.001
Discourse × Anxiety	1	58.00	89.09	< 0.001

#### Integrated interpretation of strategy models

The bottom-up models mirrored the top-down models, consistent with the near-reciprocal correlations between the two strategy indices within each discourse condition (information-rich: *r* = − .995; rhetorical: *r* = − .991; Table 2). In substantive terms, discourse type strongly cued a strategic re-weighting (information-rich toward top-down, rhetorical toward bottom-up), while Vocabulary Score and Listening Anxiety moderated the magnitude of this shift as reflected in the significant Discourse × Vocabulary and Discourse × Anxiety terms in the strategy models (Tables 6 and 7).

## Summary of hypothesis tests

Across primary paired comparisons and covariate-adjusted sensitivity models, discourse type showed a consistent and statistically robust association with listening outcomes. The information-rich discourse was associated with higher comprehension accuracy and faster task completion, alongside markedly lower perceived cognitive load. Strategy endorsement exhibited a pronounced crossover, with discourse type strongly predicting whether listeners emphasized top-down versus bottom-up processing, and with learner differences (vocabulary and anxiety) moderating the magnitude of this strategic re-weighting in the mixed models.

Across primary paired comparisons and covariate-adjusted sensitivity models, discourse type showed a consistent and statistically robust association with listening outcomes. The information-rich discourse was associated with higher comprehension accuracy and faster task completion, alongside markedly lower perceived cognitive load. Strategy endorsement exhibited a pronounced crossover, with discourse type strongly predicting whether listeners emphasized top-down versus bottom-up processing, and with learner differences (vocabulary and anxiety) moderating the magnitude of this strategic re-weighting in the mixed models. These findings should be interpreted as strong evidence for this particular sample of undergraduate EFL learners performing these specific listening tasks, rather than as unrestricted claims about all L2 listeners or all discourse genres.

This study examined whether discourse affordances shape L2 listening outcomes by contrasting an information-rich scientific passage with a rhetorically complex literary passage and tracking three linked domains: performance (accuracy and processing speed), perceived cognitive load, and metacognitive strategy emphasis. Across the paired comparisons and the covariate-adjusted sensitivity models, discourse type emerged as a robust determinant of how successfully and efficiently listeners constructed meaning, how effortful they experienced the task, and how they regulated comprehension through strategic re-weighting. In this sense, the findings are consistent with prior behavioral evidence supporting the Information-Rich Discourse Hypothesis [10], while also refining its scope by showing that learner differences (lexical resources and listening anxiety) systematically account for variance in effort and strategy reallocation.

This study examined whether discourse affordances shape L2 listening outcomes by contrasting an information-rich scientific passage with a rhetorically complex literary passage and tracking three linked domains: performance (accuracy and processing speed), perceived cognitive load, and metacognitive strategy emphasis. Across the paired comparisons and the covariate-adjusted sensitivity models, discourse type emerged as a robust determinant of how successfully and efficiently listeners constructed meaning, how effortful they experienced the task, and how they regulated comprehension through strategic re-weighting. In this sense, the findings are consistent with prior behavioral evidence supporting the Information-Rich Discourse Hypothesis [10], but they are also compatible with alternative accounts centered on predictive processing, discourse coherence, and effortful resource allocation. The results therefore strengthen the focal hypothesis while also suggesting that several contemporary frameworks converge on a similar explanation of why discourse structure matters. Learner differences (lexical resources and listening anxiety) systematically accounted for variance in effort and strategy reallocation.

The most stable finding was the size and consistency of the discourse effect on comprehension outcomes. Listeners achieved higher accuracy and completed the task more quickly in the information-rich condition than in the rhetorical condition. This pattern aligns with the view that discourse type functions as “predictive scaffolding” rather than a superficial genre label: when macro-organization is more visible and propositional development is stable, listeners can stabilize meaning earlier and allocate resources toward semantic integration rather than structural recovery [7]. The role of discourse signaling cues is particularly relevant here. Texts that provide explicit connective and summarizing markers reduce the organizational burden of tracking topic development, enabling more efficient integration of incoming propositions into a coherent situation model [6]. The performance advantage for the scientific passage is therefore interpretable as an input–processing alignment effect: the discourse affords earlier global coherence, reducing the need for costly reanalysis and repair during online comprehension.

The covariate-adjusted sensitivity model for accuracy further clarifies this picture. Discourse type remained a significant predictor of accuracy even after controlling for Vocabulary Score and Listening Anxiety, indicating that the performance gap was not simply a by-product of individual differences. At the same time, both covariates were strong predictors of accuracy, which supports the broader position that listening comprehension reflects an interaction among input characteristics and listener resources rather than a single unitary “listening ability” [7]. Importantly, the Discourse × Vocabulary and Discourse × Anxiety terms were not significant for accuracy in the mixed model, suggesting that the discourse advantage in correctness was relatively consistent across learners, even though learners differed in overall level of performance.

The covariate-adjusted sensitivity model for accuracy further clarifies this picture. Discourse type remained a significant predictor of accuracy even after controlling for Vocabulary Score and Listening Anxiety, indicating that the performance gap was not simply a by-product of individual differences. At the same time, both covariates were strong predictors of accuracy, which supports the broader position that listening comprehension reflects an interaction among input characteristics and listener resources rather than a single unitary ‘listening ability’ [7]. Importantly, the Discourse x Vocabulary and Discourse x Anxiety terms were not significant for accuracy in the mixed model, suggesting that the discourse advantage in correctness was relatively consistent across learners **within this sample of Beni-Suef University EFL majors and within the two discourse conditions tested here.**.

The cognitive load findings provide a process-oriented explanation for the performance pattern. Participants reported substantially lower load for the information-rich passage than for the rhetorical passage, and this discourse effect remained significant in the covariate-adjusted model. Contemporary accounts of listening emphasize that comprehension in time-limited speech requires continual allocation of processing resources to segmentation, lexical access, integration, and repair; when the input increases ambiguity or reduces predictability, resource demands rise because listeners must sustain multiple candidates, re-evaluate local interpretations, and re-establish coherence [2]. The rhetorical passage operationalized precisely these pressure points—interpretive openness, less transparent progression, and higher likelihood of local breakdown—so the observed load increase is consistent with a resource reallocation response to input uncertainty [2, 18].

The cognitive load findings provide a process-oriented explanation for the performance pattern. Participants reported substantially lower load for the information-rich passage than for the rhetorical passage, and this discourse effect remained significant in the covariate-adjusted model. Contemporary accounts of listening emphasize that comprehension in time-limited speech requires continual allocation of processing resources to segmentation, lexical access, integration, and repair; when the input increases ambiguity or reduces predictability, resource demands rise because listeners must sustain multiple candidates, re-evaluate local interpretations, and re-establish coherence [2]. The rhetorical passage operationalized precisely these pressure points - interpretive openness, less transparent progression, and higher likelihood of local breakdown - so the observed load increase is consistent with a resource reallocation response to input uncertainty [2, 18]. At the same time, because cognitive load was measured through immediate self-report rather than behavioral or physiological indices, the present findings should be read as evidence about perceived effort rather than a full multimethod account of objective processing load.

### Metacognitive regulation as discourse-contingent strategy re-weighting

The strategy results offer the clearest behavioral trace of adaptive regulation. Listeners reported greater top-down emphasis for the information-rich passage and greater bottom-up emphasis for the rhetorical passage, producing a pronounced crossover pattern. This is congruent with models in which metacognition is not a fixed trait but a situated response: when discourse structure is visible and meaning is stabilizing, learners can invest in proactive processes such as prediction, inferencing, and macro-integration; when the input is rhetorically dense and less predictable, learners shift toward reactive processes such as lexical checking, syntactic parsing, and repeated local verification [11]. The shift therefore supports the Input–Processing–Regulation view articulated in the theoretical framework: discourse affordances (input) constrain resource allocation (processing), which triggers strategic re-weighting (regulation) that ultimately shapes comprehension outcomes.

Crucially, the covariate-adjusted sensitivity models revealed that strategy re-weighting was moderated by both Vocabulary Score and Listening Anxiety. For top-down use, Discourse × Vocabulary and Discourse × Anxiety interactions were significant; the same was true for bottom-up use. These moderation effects indicate that the *magnitude* of strategic reallocation depends on learner resources and affective state. Learners with stronger lexical resources appear better positioned to sustain or recover higher-level integration when discourse affordances weaken, whereas learners with higher anxiety appear to shift more sharply toward local checking under rhetorical demands, consistent with an effortful compensation pattern under uncertainty [5]. This extension is important because it refines the hypothesis from a purely discourse-driven shift to an interactive model in which discourse cues the direction of strategic change, while learner characteristics help determine how strongly that change manifests [7].

### Interpreting the top-down / bottom-up “mirror” pattern

A methodological issue emerged in the strategy measures: top-down and bottom-up indices were nearly perfectly negatively correlated within each discourse condition. This pattern suggests that, in this dataset, the two scales functioned less like independent constructs and more like opposite poles of a single strategic emphasis dimension. Substantively, that does not undermine the central finding that listeners re-weight strategy emphasis across discourse types; rather, it narrows what can be claimed. The most defensible interpretation is that discourse type cued a shift in *relative strategic emphasis* (from global meaning integration toward local decoding and verification, or vice versa), but the data do not support treating top-down and bottom-up as fully separable latent variables in this measurement configuration. This limitation strengthens the case for future work to adopt instruments and designs that better preserve construct independence and capture real-time monitoring behavior more directly [11].

### Role of vocabulary and construct validity

Vocabulary was a powerful covariate in the mixed models, but its interpretation must match what was actually measured. The Vocabulary Score in this study indexed receptive vocabulary knowledge via a written-format measure [9]. It therefore serves most appropriately as a proxy for lexical coverage and general lexical resources rather than as evidence of phonological vocabulary access or lexical automatization during speech processing. Accordingly, the vocabulary effects should be interpreted conservatively: learners with higher receptive vocabulary likely experienced fewer lexical gaps and therefore reduced reliance on compensatory inference, which supports more stable integration and lower effort, especially under rhetorically demanding input [9]. Claims about “phonological efficiency” or automatized spoken-word recognition cannot be made on the basis of the present vocabulary measure, and the implications of this construct mismatch should be explicitly reflected in the limitations and in recommendations for future measurement.

## Limitations

The limitations can be organized into three categories to clarify the scope of the findings. Methodological limitations: the study contrasted two intentionally distant discourse conditions and used a within-subject laboratory task with one exposure per passage. This design increased leverage for detecting discourse effects, but it also means that the findings should not be generalized automatically to intermediate discourse forms (e.g., lectures, news reports, or conversational narratives), repeated-listening contexts, or substantially different instructional settings.

Measurement limitations: both cognitive load and strategy use relied primarily on self-report, and no complementary behavioral or physiological measures (e.g., pupillometry, eye-tracking, or response-locked process tracing) were available. Consequently, the study provides strong evidence about perceived effort and reported strategic emphasis, but not a fully triangulated account of objective processing dynamics. In addition, the top-down and bottom-up indices were extremely negatively coupled in this dataset, which limits claims about their construct independence.

Sampling and construct-alignment limitations: the sample comprised 60 undergraduate EFL majors from one university, so the results are best interpreted as applying to learners with roughly comparable proficiency, educational background, and task experience rather than to all EFL populations. Vocabulary was included as an individual-difference covariate because lexical coverage plausibly supports word recognition and proposition building during continuous speech. However, the measure used here - the updated Vocabulary Levels Test - indexes written receptive vocabulary knowledge and therefore should be interpreted as a proxy for general lexical resources/coverage rather than as a direct assessment of phonological vocabulary access or lexical automatization in listening [9].

Accordingly, any covariate effects involving vocabulary should be read as reflecting broad lexical resources that may facilitate comprehension and reduce perceived effort, not as evidence about the efficiency of spoken-form access. Future studies should strengthen construct alignment by using an aural lexical measure (e.g., a listening vocabulary levels test) and/or an automatization-sensitive lexical access task alongside written vocabulary indices. They should also incorporate multimethod effort measures and more diverse participant pools so that claims about discourse effects can be evaluated across a wider range of L2 listeners and task ecologies.

## Pedagogical implications and future directions

The discourse effects have immediate implications for listening pedagogy. The performance and load gap suggests that learners benefit from instruction that makes macro-organization “visible,” including explicit teaching of discourse signaling cues and rhetorical moves that support global integration [6]. For rhetorically complex or literary input, the results suggest that reducing avoidable uncertainty—through schema activation, guided preview of key propositions, and structured post-listening integration—may be essential to prevent excessive load escalation and to enable listeners to maintain meaning-level processing rather than collapsing into purely local decoding [18]. The moderation results also imply differentiated support: learners with lower lexical resources or higher anxiety may require stronger scaffolding to manage rhetorical discourse without disproportionately increasing effort and reactive checking [5, 9].

The discourse effects have immediate implications for listening pedagogy. The performance and load gap suggests that learners benefit from instruction that makes macro-organization ‘visible,’ including explicit teaching of discourse signaling cues and rhetorical moves that support global integration [6]. For rhetorically complex or literary input, the results suggest that reducing avoidable uncertainty - through schema activation, guided preview of key propositions, and structured post-listening integration - may be essential to prevent excessive load escalation and to enable listeners to maintain meaning-level processing rather than collapsing into purely local decoding [18]. The moderation results also imply differentiated support: learners with lower lexical resources or higher anxiety may require stronger scaffolding to manage rhetorical discourse without disproportionately increasing effort and reactive checking [5, 9]. These recommendations are directed most directly to advanced university-level EFL contexts similar to the present sample, and they should be transferred to other learner groups only with appropriate caution.

## Conclusion

By contrasting an information-rich scientific discourse with a rhetorically complex literary discourse, this study provides converging evidence that discourse affordances shape L2 listening in three linked domains: outcome success (accuracy), efficiency (speed), and processing cost (cognitive load). Information-rich discourse—characterized by clearer organization and more explicit cues—was associated with higher accuracy, faster completion, and substantially lower perceived effort. Rhetorical discourse—characterized by interpretive openness and reduced predictability—was associated with poorer performance, slower processing, and heightened effort, alongside a strong shift toward bottom-up strategic emphasis.

By contrasting an information-rich scientific discourse with a rhetorically complex literary discourse, this study provides converging evidence that discourse affordances shape L2 listening in three linked domains: outcome success (accuracy), efficiency (speed), and processing cost (cognitive load). Information-rich discourse - characterized by clearer organization and more explicit cues - was associated with higher accuracy, faster completion, and substantially lower perceived effort. Rhetorical discourse - characterized by interpretive openness and reduced predictability - was associated with poorer performance, slower processing, and heightened effort, alongside a strong shift toward bottom-up strategic emphasis. These conclusions are most defensible for the present sample of undergraduate EFL learners completing the specific tasks used in this study, not as universal claims about all listeners or all forms of literary and scientific discourse.

At the same time, the study refines the Information-Rich Discourse Hypothesis by demonstrating that strategic adaptation is not only discourse-cued but also moderated by listener characteristics. Vocabulary (as receptive lexical resources) and listening anxiety accounted for substantial variance in outcomes and effort, and they shaped the magnitude of strategy re-weighting across discourse types. Taken together, the findings support an interactional view of L2 listening in which comprehension emerges from the coupling of discourse affordances, learner resources, and metacognitive regulation [7, 10]. They also suggest that the Information-Rich Discourse Hypothesis is strengthened when interpreted alongside complementary frameworks of predictive processing, discourse coherence, and resource allocation rather than in isolation. This provides a more precise basis for theory-building and for pedagogical approaches that explicitly target discourse navigation, effort management, and adaptive strategy use under rhetorically demanding listening conditions.

## Supplementary Information

Supplementary Material 1.

Supplementary Material 2.

Supplementary Material 3.

Supplementary Material 4.

Supplementary Material 5.

Supplementary Material 6.

Supplementary Material 7.

## Data Availability

The datasets generated and analyzed during the current study are available as electronic files attached to this submission.

## Abbreviations

CEFR: Common European Framework of Reference for Languages
CLT: Cognitive Load Theory
EFL: English as a Foreign Language
L2: Second language
LMM: linear mixed model
MSI: Metacognitive Strategy Inventory
NVLT: New Vocabulary Levels Test
WM: Working memory
