# Supplementary material for: Discourse type effects on EFL listening comprehension: cognitive load and metacognitive strategy shifts across scientific and literary texts
Source: BMC Psychol. 2026 May 13;14:708. doi: 10.1186/s40359-026-04684-2 (PMC13169529; doi:10.1186/s40359-026-04684-2)
Supplement: Supplementary file 7 — Supplementary Material 7. [file 40359_2026_4684_MOESM7_ESM.docx]

**Research Instruments**

**Part 1: Listening Stimuli (Audio Scripts)**

**Audio Passage A: Information-Rich (Scientific)**

**Title:** The Unseen Guardian: Understanding Mangrove Ecosystems
**Approximate Reading Time:** 4 minutes
**Word Count:** 581 words

**(Start of Audio Script)**

"Mangrove forests are one of the planet's most vital coastal ecosystems. Found along tropical and subtropical coastlines, these unique forests of salt-tolerant trees appear as dense, tangled jungles thriving at the edge of the sea. Their dense network of prop roots, which arch above the water, serves several critical functions that support both environmental stability and biodiversity.

First, and most visibly, these roots are masters of coastal engineering. They trap vast amounts of sediment flowing from rivers, which helps to build up and stabilize the coastline, preventing erosion from waves and tides. This process makes them a powerful natural barrier. Specifically, scientific studies have shown that a healthy mangrove forest can reduce the destructive force of storm surges and even tsunami waves, with some estimates suggesting they can reduce wave height by up to 66%. This natural defense system protects vulnerable coastal communities, offering a level of security that man-made structures often cannot match. Furthermore, by anchoring the soil, they prevent valuable coastal land from being washed out to sea.

Second, the complex underwater root system provides a critical nursery habitat for countless species of marine life. In addition to the fish, crabs, and shellfish mentioned in many textbooks, these habitats are crucial for the early life stages of commercially important species like shrimp and groupers. The tangled roots offer young animals protection from larger predators, while the nutrient-rich water, enriched by decaying leaf litter, provides an abundant food source. This creates what scientists call a 'biodiversity hotspot,' a place where marine life can thrive. The health of many offshore fisheries, including valuable coral reefs, is directly linked to the health of these coastal mangrove nurseries.

A third, and perhaps most critical, role of mangroves in our modern world is their incredible capacity for carbon sequestration. Mangrove soils are highly effective at capturing and storing carbon dioxide from the atmosphere, a process known as 'blue carbon.' In fact, on a per-hectare basis, mangrove forests can store up to ten times more carbon than terrestrial forests. This makes their preservation a crucial strategy in the global effort to mitigate climate change. By locking carbon away in their waterlogged soil for centuries, they prevent it from entering the atmosphere as a greenhouse gas.

Unfortunately, despite their immense value, these crucial ecosystems are under severe threat. The primary drivers of deforestation are coastal urban development and the expansion of aquaculture, particularly shrimp farming, which involves clearing large tracts of mangrove forest to create ponds. This destruction not only removes the protective barrier for coastal towns and devastates local fisheries, but it also releases massive amounts of stored carbon back into the atmosphere. In summary, mangrove forests are not just coastal woodlands; they are multifaceted ecosystems that provide coastal protection, support biodiversity, and combat climate change. Therefore, their conservation is not just an environmental issue, but an economic and social imperative for coastal regions worldwide."

**(End of Audio Script)**

**Audio Passage B: Rhetorical (Literary)**

**Title:** An Analysis of Hamlet's Soliloquy: 'To Be, or Not to Be'
**Approximate Reading Time:** 4 minutes
**Word Count:** 572 words

**(Start of Audio Script)**

"William Shakespeare's tragedy, *Hamlet*, is perhaps the most famous play in the English language, and at its heart lies a soliloquy of unparalleled philosophical depth and rhetorical complexity: the 'To be, or not to be' speech. Delivered by Prince Hamlet in Act Three, the speech is not a simple monologue, but a profound internal debate. The prince, tormented by his father's murder and his mother's hasty marriage to the killer, contemplates the ultimate question of existence versus non-existence. To understand its power, we will first listen to a dramatic reading of the complete soliloquy, and then briefly analyze its complex structure and meaning.

**(Dramatic Reading Section)**
To be, or not to be, that is the question:
Whether 'tis nobler in the mind to suffer
The slings and arrows of outrageous fortune,
Or to take arms against a sea of troubles
And by opposing end them. To die—to sleep,
No more; and by a sleep to say we end
The heart-ache and the thousand natural shocks
That flesh is heir to: 'tis a consummation
Devoutly to be wish'd. To die, to sleep;
To sleep, perchance to dream—ay, there's the rub:
For in that sleep of death what dreams may come,
When we have shuffled off this mortal coil,
Must give us pause—there's the respect
That makes calamity of so long life.
For who would bear the whips and scorns of time,
Th'oppressor's wrong, the proud man's contumely,
The pangs of dispriz'd love, the law's delay,
The insolence of office, and the spurns
That patient merit of th'unworthy takes,
When he himself might his quietus make
With a bare bodkin? Who would fardels bear,
To grunt and sweat under a weary life,
But that the dread of something after death,
The undiscovere'd country, from whose bourn
No traveller returns, puzzles the will,
And makes us rather bear those ills we have
Than fly to others that we know not of?
Thus conscience does make cowards of us all,
And thus the native hue of resolution
Is sicklied o'er with the pale cast of thought,
And enterprises of great pith and moment
With this regard their currents turn awry,
And lose the name of action.

**(Narrator's Analysis Section)**
As we can hear, Hamlet's language is dense with metaphor and complex syntax. He frames life's hardships as 'slings and arrows' and 'a sea of troubles,' transforming abstract suffering into physical assaults. The central metaphor, comparing death to a 'sleep,' is immediately complicated by a terrifying uncertainty: the possibility of dreams. This is what he calls 'the rub'—the obstacle. The phrase 'mortal coil' refers to the turmoil and bustle of mortal life, which we would 'shuffle off' in death.

Hamlet's argument is that the fear of the unknown—the 'undiscovere'd country' from which no one returns—is what forces humanity to endure a life filled with injustice and pain. He lists these pains vividly: the oppression, the arrogance of the powerful, the sting of unrequited love, and the frustration of delayed justice. This fear of the afterlife, he concludes, paralyzes our will. Our 'conscience,' meaning our consciousness or inward thought, makes us cowards. It transforms our bold resolutions into pale inaction. In this speech, Hamlet is not merely contemplating his own suicide; he is dissecting the universal human condition, where the terror of the unknown often outweighs the certainty of present suffering."

**(End of Audio Script)**

**Part 2: Instrument 1: Listening Comprehension Tests**

**General Instructions:** "After listening to the audio, you will answer 10 questions. Please read each question carefully. Your accuracy and response time will be recorded."

**Test A: Comprehension Test for Mangrove Ecosystems**

**Section 1: Literal Comprehension**

1. What specific term does the speaker use for the process of carbon storage in mangrove soils?
   a) Green carbon
   b) Soil sequestration
   c) Blue carbon
   d) Marine trapping
2. The main threats to mangroves mentioned are urban development and what other industry?
   a) Commercial logging
   b) Shrimp farming
   c) Oil drilling
   d) Tourism

**Section 2: Inferential Comprehension**
3. The passage suggests that a decline in mangrove forests would likely lead to:
a) A decrease in coastal flooding and erosion.
b) An increase in the populations of commercially important fish.
c) A negative impact on local fishing economies.
d) An improvement in water quality for coastal cities.

1. The speaker describes mangrove conservation as a "social imperative." This implies that protecting mangroves is:
   a) Only important for environmental activists.
   b) Beneficial for the safety and well-being of human communities.
   c) A low-priority issue compared to economic development.
   d) A responsibility that belongs only to scientists.

**Section 3: Global Comprehension**
5. In one sentence, what is the central message of this audio passage?
*(Short Answer)*

**Test B: Comprehension Test for Hamlet's Soliloquy**

**Section 1: Literal Comprehension**

1. According to the narrator, what does the phrase "mortal coil" refer to?
   a) The physical body after death.
   b) A type of ancient weapon.
   c) The turmoil and bustle of life.
   d) A feeling of guilt or conscience.
2. Hamlet refers to the afterlife as the "undiscovere'd country" because:
   a) He believes it is a beautiful and peaceful place.
   b) No one has ever returned from it to describe what it is like.
   c) It is a place that only exists in dreams.
   d) He is planning a long journey there.

**Section 2: Inferential Comprehension**
3. The narrator's analysis suggests that Hamlet's hesitation to act comes from:
a) His physical inability to fight his enemies.
b) His hope that his situation will improve on its own.
c) His profound fear of the uncertainty of death.
d) His desire to follow the law.

1. The line "conscience does make cowards of us all" most likely means that:
   a) Only guilty people are cowards.
   b) Overthinking and fear of the unknown prevent bold action.
   c) Society's rules stop people from being free.
   d) It is cowardly to feel sorry for oneself.

**Section 3: Global Comprehension**
5. In one sentence, what is the main internal conflict that Hamlet is exploring in the soliloquy?
*(Short Answer)*

**Part 3: Instrument 2: Self-Reported Cognitive Load Scale**

**Instructions:** "Please reflect on the listening task you just completed. On a scale of 1 to 7, please rate your experience for each statement."
*(Administered immediately after each comprehension test)*

1. **How much mental effort did you invest in understanding this audio?**
   1 (Very Low Effort) --- 2 --- 3 --- 4 --- 5 --- 6 --- 7 (Very High Effort)
2. **How difficult did you find this listening passage?**
   1 (Very Easy) --- 2 --- 3 --- 4 --- 5 --- 6 --- 7 (Very Difficult)
3. **How confusing or frustrating was this listening passage?**
   1 (Not at all Frustrating) --- 2 --- 3 --- 4 --- 5 --- 6 --- 7 (Extremely Frustrating)

**Part 4: Instrument 3: Metacognitive Strategy Inventory (MSI)**

**Instructions:** "Please think back to the two audio passages: the one about **Mangroves** and the one about **Hamlet**. Rate how frequently you used each strategy below for each text."

| **Listening Strategy** | **For the Mangrove Text** | **For the Hamlet Text** |
| --- | --- | --- |
| *(Scale: 1=Never, 2=Rarely, 3=Sometimes, 4=Often, 5=Very Frequently)* |  |  |
| **Top-Down Strategies (Focus on Meaning)** |  |  |
| 1. I used my background knowledge to understand the topic. | 1--2--3--4--5 | 1--2--3--4--5 |
| 2. I focused on the main ideas and the overall message. | 1--2--3--4--5 | 1--2--3--4--5 |
| 3. I connected ideas to form a mental summary as I listened. | 1--2--3--4--5 | 1--2--3--4--5 |
| **Bottom-Up Strategies (Focus on Form)** |  |  |
| 4. I concentrated on understanding the meaning of every single word. | 1--2--3--4--5 | 1--2--3--4--5 |
| 5. I had to mentally re-listen to or analyze a difficult sentence structure. | 1--2--3--4--5 | 1--2--3--4--5 |
| 6. I had to mentally translate specific words or phrases into my native language. | 1--2--3--4--5 | 1--2--3--4--5 |

**Part 5: Instrument 4: Learner Background and Proficiency Profile**

**Instructions:** "Before we begin the main tasks, please provide the following information. All data will be kept confidential and anonymous."

**Section A: Background Information**

- Age: ________
- Native Language: ________
- Years of formal English study: ________
- Please self-assess your English proficiency (CEFR): (Circle one) B1 | B2 | C1 | C2

**Section B: Listening Confidence and Anxiety Scale**
*Instructions: Please indicate your agreement with the following statements.*
*(Scale: 1=Strongly Disagree, 2=Disagree, 3=Neutral, 4=Agree, 5=Strongly Agree)*

1. I generally feel confident when listening to academic English. (1--2--3--4--5)
2. I become anxious when I hear complex sentences or unfamiliar vocabulary. (1--2--3--4--5)

**Section C: Vocabulary Size Test**
*Instructions: This final section is a standardized test to provide a baseline measure of your English vocabulary knowledge. The test is divided into several sections based on how common the words are in English.*

*In each section, you will see a set of words and a set of definitions. Your task is to match each word to its correct definition. Please work as accurately as you can. There is no time limit, but please proceed at a steady pace. This is not a test of your memory of the audio passages; it is a general vocabulary test.*

**Part 1: Example from the 2,000-word Level**

*Instructions: For each word on the left, find the correct definition on the right. Write the letter of the correct definition in the space provided.*

| **Word** | **Definition** |
| --- | --- |
| 1. industry ______ | a. to keep someone or something safe from harm or injury |
| 2. nature ______ | b. the physical world, including plants, animals, and landscapes, separate from humans |
| 3. protect ______ | c. to grow or change into something more advanced or stronger |
| 4. develop ______ | d. the production of goods in factories |
|  | e. the money used in a particular country |
|  | f. a strong feeling of dislike |

**Part 2: Example from the 3,000-word Level**

*Instructions: For each word on the left, find the correct definition on the right. Write the letter of the correct definition in the space provided.*

| **Word** | **Definition** |
| --- | --- |
| 1. ecosystem ______ | a. a group of people who live in the same area or have a shared characteristic |
| 2. community ______ | b. a formal discussion where different opinions are expressed |
| 3. strategy ______ | c. all the living things in an area and how they affect each other and the environment |
| 4. debate ______ | d. a detailed plan for achieving success in a situation like war, politics, or business |
|  | e. a formal agreement between two or more countries |
|  | f. the basic systems and services that a country or organization uses |

**Part 3: Example from the 5,000-word Level (Academic Vocabulary)**

*Instructions: For each word on the left, find the correct definition on the right. Write the letter of the correct definition in the space provided.*

| **Word** | **Definition** |
| --- | --- |
| 1. philosophy ______ | a. the protection of animals, plants, and natural resources |
| 2. metaphor ______ | b. a statement that seems contradictory but may in fact be true |
| 3. conservation ______ | c. the study of the fundamental nature of knowledge, reality, and existence |
| 4. paradox ______ | d. a figure of speech in which a word or phrase is applied to something to which it is not literally applicable |
|  | e. an official examination of accounts or records |
|  | f. a system in which people or things are arranged according to their importance |

**Concluding Note for the Participant:**
"The actual test will contain several more sets of words like these, progressing in difficulty. Please continue until the test indicates you are finished. Thank you for your concentration."

**Scoring Protocol (for researcher use)**

- **Administration:** The vocabulary measure was delivered as a computer-based matching task organized by frequency bands (2,000; 3,000; 4,000; 5,000 levels). Items were presented in sets, with participants matching words to brief definitions.
- **Item sourcing:** Target words were sampled from established frequency-based word lists (e.g., BNC/COCA-based lists). The level structure and matching-task logic follow common vocabulary-levels testing principles used in vocabulary assessment research (e.g., Webb et al., 2017), but the present instrument was **researcher-compiled** and **does not reproduce** any published test forms.
- **Scoring:** Responses were scored automatically. A continuous **Vocabulary Score** was computed as the **percentage of correct matches** across all levels combined. Level-wise subscores were retained for screening and descriptive checks.
- **Use in analyses:** The Vocabulary Score was used as a covariate to control for baseline differences in receptive lexical resources.
